# Understanding the challenges faced by Michigan’s family farmers: race/ethnicity and the impacts of a pandemic

**DOI:** 10.1007/s10460-022-10305-6

**Published:** 2022-03-03

**Authors:** Dorceta E. Taylor, Lina M. Farias, Lia M. Kahan, Julia Talamo, Alison Surdoval, Ember D. McCoy, Socorro M. Daupan

**Affiliations:** 1 School of the Environment, Yale University, 195 Prospect Street, New Haven, CT 06511 USA; 2 14 E Cache La Poudre St, Colorado Springs, CO 80903 USA; 3 The College of Wooster, 1189 Beall Ave, Wooster, OH 44691 USA; 4 The Nature Conservancy, 4245 North Fairfax Drive, #100, Arlington, VA 22203 USA; 5 School for the Environment and Sustainability, University of Michigan, 440 Church Street, Ann Arbor, MI 48109 USA; 6 Clean Water Action, 1315 Walnut Street, Philadelphia, 19107 USA

**Keywords:** COVID, Loans, Financial insecurity, People of Color, Whites, Organic, Food insecurity, Food assistance, Gleaning, Online

## Abstract

Michigan is a critical agricultural state, and small family farms are a crucial component of the state’s food sector. This paper examines how the race/ethnicity of the family farm owners/operators is related to farm characteristics, financing, and impacts of the pandemic. It compares 75 farms owned/operated solely by Whites and 15 with People of Color owners/operators. The essay examines how farmers finance their farm operations and the challenges they face doing so. The article also explores how the Coronavirus-19 (COVID-19) pandemic affected farming operations, the financial viability of farms, and how farmers responded to the challenges posed by the pandemic. The study found that People of Color farm owners/operators were younger than White farm owners/operators. The People of Color farm owners/operators tended to manage smaller farms for shorter periods than White farm owners/operators. Though two-thirds of the Farmers of Color owned their farms, they were more financially vulnerable than White farm owners/operators. The farmers studied had difficulty obtaining loans to finance their farms. Farmers reported increasing requests from people for food assistance during the pandemic. Farmers responded to the pandemic by participating in government programs such as the Farm to Families Food Box Program that purchased their produce. It allowed farmers to supply emergency food assistance programs with products from their farms. The products went to families receiving food assistance from soup kitchens, food banks, and other community-based nonprofits.

## Introduction

Despite the consolidation of farms and a shift to more extensive farming operations over the past three decades, most of the farms in the U.S. are still small, family-run operations. That is, 89.9% of the farms in America are considered small. Small farms average 231 acres in size and are typically family operated^1^ (MacDonald et al. 2018; Hoppe 2017; Carlisle et al. 2019; Dunckel 2013). The U.S. Department of Agriculture (USDA) defines a farm as small if it has gross cash farm income under $350,000^2^ (Whitt 2021; MacDonald et al. 2018; Hoppe 2017). This research article examines family farm operators in Michigan because the state has a significant agricultural sector. These types of farms play vital roles in food production in the state. In the U.S., 76% of farms have annual sales below $50,000. Moreover, 58% of the farms had sales of less than $10,000 annually (USDA 2019d). In Michigan, 76% have lower sales than $50,000, and 57% of the farms have less than $10,000 in sales (USDA 2019a).

The 2017 Census of Agriculture reports that 47,641 farms occupy roughly 9.8 million acres in Michigan. The average size of those farms is 205 acres. Like the rest of the country, Michigan is losing its farms. Nationwide, the number of farms fell by 3.2% between 2012 and 2017. However, Michigan—which had 56,014 farms in 2007—lost 17.6% of those farms a decade later (USDA 2019a, b).

It is also vital to study farms and farm owners/operators because the USDA reports that farmers are experiencing rising financial insecurity. Studies show that since 2012, farm sales have declined, and farm debt has increased across the country. It is particularly true of small farms. When profit margins are examined, the status of more than half of all small farms is considered a high or medium risk for financial problems (Key et al. 2019; Hoppe 2017; Carlisle et al. 2019).

Farmers as so financially stressed that studies show that many need to have off-farm jobs to make ends meet (USDA 2019d; Carlisle et al. 2019). The value of the products sold by Michigan’s farmers was $8.2 billion in 2017; however, the amount of the sales was 5% lower than it was in 2012. Over the same period, government assistance to farmers had increased by 7%.

Farm production expenses had also risen by 4% (USDA 2019a). Michigan produces one of the most diverse sets of crops in the nation (Matts et al. 2015). The state ranks 18th overall in the sale of farm produce, 15th in the production of crops, and 21st in livestock production. Michigan is the fifth largest producer of fruits, tree nuts, and berries and the sixth-largest producer of nursery, greenhouse, floriculture crops, and sod. The state ranks seventh in milk production from cows and tenth in vegetable, melon, potato, and sweet potato production.

Michigan is also the 11th largest producer of hogs and pigs in the country (USDA 2019a).

People from a variety of backgrounds farm in Michigan. Ergo, this paper examines how the race/ethnicity of the family farm owners/operators is related to farm characteristics, financing, and impacts of the pandemic. The essay examines how farmers finance their farm operations and the challenges they face in doing so. It compares farms operated by Whites and People of Color in the state. The article is one of the first to explore how the Coronavirus-19 (COVID-19) pandemic affected farming operations, the financial viability of farms, and how farmers responded to the challenges posed by the pandemic.

Why study the relationship between race/ethnicity and farm ownership and operation? Race/ethnicity is an essential factor influencing becoming a farmer and being successful at it. Nationally, White farm owners generate 98% of all farm-related income. Typically, Farmers of Color own less land and generate much smaller incomes than White farmers. Farmers of Color are also more likely to rent or lease the farms they operate than White farmers (Carlisle et al. 2019; Horst and Marion 2019). There is ample documentation that the USDA systematically discriminated against Blacks, Native Americans, Latinx, and women farmers by denying them credit to operate their farms (Taylor 2018; Daniel 2013; Minkoff-Zern and Sloat 2017; Carlisle et al. 2019). Consequently, this first-of-a-kind study examines People of Color owned/operated farms to provide vital information about their practices and viability in the pandemic era.

## The demographic characteristics of Michigan’s farmers

The state’s farming sector is predominantly White. Hence, 98.2% of the farmers in the state are White. Black farmers in Michigan account for 0.4% of the farmers, Native Americans constitute 0.4%, Asians comprise 0.2%, and Latinx farmers make up 1.3% of the farmers (USDA 2019a, b, c; White and King 2019). Michigan’s farmers are also overwhelmingly male; 64.8% are male, and 35.3% are female. Michigan mirrors the national trend; 36% of all producers nationwide are female. The national average age of farmers is 57.5 years of age. Michigan’s farmers also tend to be older than the rest of the state’s population; 31.2% are 65 years and older, 59.8% are between 35 and 64 years old, and a mere 9% are less than 35 years old.

A 2012 study of 311 of Michigan’s organic farmers found that 85% were male. This study found that 28% of the farmers were 65 years or older (Matts et al. 2015). Miller and McCole (2014) studied 37 farmers in Southeast Michigan and found that 74.3% were male. The researchers also found that 19% of the farmers in their study were 60 years and older.

Studies of Michigan’s farmers usually ignore how the race or gender of the farm operators influence action (see, for example, Montri et al. 2021). However, one study that examines 32 female farmers in Michigan’s Lower Peninsula assessed the relationship between women, farming, and empowerment (Wright and Annes 2016).

A recent study of 27 farmers in Michigan shows that 59% of them own their farm, while 11% rent, lease, or borrow the land they farm (Montri et al. 2021). Scholars also report that some of Michigan’s farms struggle financially (Black and Jones 2019). Data showing that 37% of farmers in Montri et al.’s (2021) study rely on off-farm income to support their businesses lend credence to this claim.

The financial insecurity in Michigan’s farming sector is troubling. Studies have shown that Farmers of Color in Michigan experience discrimination in gaining access to farmlands and obtaining loans and grants to finance their farms (Taylor 2018; Tyler et al. 2014). Consequently, the paper assesses if and how Farmers of Color can finance their farms and cope with the disruptions that the pandemic caused.

### Farmers and government’s response to COVID-19

The pandemic has had far-reaching impacts on farm owners/operators. Benton (2020), Dickinson (2020), and Emmad and Peña (2020) argue that the pandemic laid bare the fragility of the food system as disruptions in the supply chain created food shortages in some places. Moreover, farmers had a surplus of milk or other foods on their hands because restaurants, schools, and colleges closed. The pandemic also meant that farmers had to decide how early they could start working in their fields, how many people they could hire, what to grow or produce, and where and how to process the products from their farms. Consequently, Blay-Palmer et al. (2020) predict that the pandemic will transform the food system because of the adjustments farmers and other food actors make in response to COVID-19. For example, the researchers argue that many food producers will move some of their operations online.

Farmers who experienced market disruptions and other costs associated with COVID-19 are eligible to apply to the Farm Service Agency (FSA) for assistance through the Coronavirus Food Assistance Program (CFAP) (Farm Service Agency 2020). In addition, the USDA approved a $4.5 billion package to connect producers with consumers through the Farmers to Families Food Box Program (FFFBP). The program helped producers sell foods originally intended for restaurants (Agricultural Marketing Service 2021; Galloway 2020). The USDA contracted with distributors and wholesalers to provide pre-packed boxes of fresh produce, dairy, and meat to food banks, faith-based organizations, and local nonprofits to distribute to families in need of food. The food boxes contained several Michigan products such as apples, asparagus, bell peppers, blueberries, bratwurst, cabbage, carrots, celery, cheese, concord grapes, cucumbers, fall squash, onions, peaches, potatoes, tomatoes, yellow squash, and zucchini (Sielski 2020). According to the Agricultural Marketing Service (2021), between May 15 and December 31, the FFFBP delivered 132.7 million boxes of food to families nationwide. In Michigan, one distributor—Eastern Market—packaged and delivered 2000 food boxes to food banks and other nonprofits weekly (Galloway 2020).

## Methodology

### Definition of farm size

We use the USDA’s definition of farm size to guide our identification and selection of farmers for our study. A farm is an agricultural operation that generates at least $1000 in annual sales. Most of the farmers included in our study operate farms with yearly revenues of less than $350,000. Hence, we classify them as small farmers (Whitt 2021; MacDonald et al. 2018; MacDonald 2017; Hoppe 2017). Only six of the farmers in the sample had farm revenues that exceeded $350,000.

Another indicator of size is the acreage of the farm. All but three of the farmers in the study operate farms that are smaller than 180 acres. A small farm is under 180 acres (North Carolina Department of Agriculture 2020). MacDonald et al. (2013) also use acreage to help classify farms. For this study, we will examine the following categories of farms: under 20 acres, farms that are 20–99 acres, and those that are 100 acres or more. We will also analyze farms with the following revenue streams: those for which the revenue was unknown, farms with incomes of $1000–$99,000, and those with revenues of $100,000 or more.

### Defining farm owners/operators and race/ethnicity

The terms farm owners, farm operators, and farmers are used interchangeably in this paper to refer to the people who own or operate the farms in the sample. Some respondents own the land they farm, while others rent, lease, or borrow land to operate farms on them. The three terms describe the primary or secondary owners/operators. We categorize a farm as owned/operated by Whites if the farm has a sole owner/operator who is White or a primary and secondary owner/operator who is White. In other words, no People of Color own/operate the farm in a primary or secondary capacity.

We categorize a farm as People of Color owned/operated if the sole owner/operator is a Person of Color. If either the farm’s primary or secondary owner/operator is also a Person of Color, we categorize the farm as a People of Color owned/operated entity. Thus, if at least one of the farm’s primary or secondary owners/operators is a Person of Color, the farm is considered Person of Color owned/operated.

### Survey methodology: identifying and selecting family farmers in Michigan

We studied family farming operations in Michigan during the summer and fall of 2020. We used the Agricultural Marketing Service (2020) directory to identify Michigan farms. We also used the following directories to identify farms: the West Michigan Growers Group, West Michigan Farms to Visit, West Michigan Farm Link, Michigan U-Pick Farms, West-Central Michigan U-Pick Farms, Discover Michigan Farm Fun Directory, Michigan Farm Bureau’s Agritourism Directory, Michigan Organic Food and Farm Alliance, and the Upper Peninsula Food & Farm Directory.

We communicated with 300 farmers we had contact information for to ask them to complete a survey about the farm they operate. The survey, designed on a Qualtrics platform, could be administered by telephone, or self-administered (Qualtrics 2020). Farmers were usually too busy during daytime hours to take a telephone survey, so we sent them a hyperlink to the survey to complete at their convenience. Farmers were offered $35 in compensation for their time if they completed the survey; it took about 45 min to complete the instrument. We surveyed from June 12, 2020, to February 5, 2021. We received 205 responses. One hundred and thirty-seven of the responses were usable. However, this study analyzes 90 farm operations that meet the outlined criteria. We downloaded data from the Qualtrics survey into SPSS 27.0 for statistical analysis (IBM 2020).

### Coding open-ended responses and identifying themes

In addition to the close-ended responses discussed throughout the paper, we discuss responses from four open-ended questions that farmers answered. Farmers who said they faced challenges in obtaining farm financing also reported what challenges they encountered.

We used the participants’ responses to create the 32 categories in Table 4. While some farmers faced single obstacles, others faced multiple challenges. We coded each unique challenge into a separate category.

We asked farmers to say what kinds of government assistance programs they gained access to during the pandemic. We used the open-ended responses to identify the government programs that assisted them. We coded each unique program that farmers listed separately (see Table 5).

Respondents indicated how they felt about government responses to farmers during the pandemic. We coded the answers into one of four categories: (a) responses that criticized the government; (b) ambivalent responses—these comments were simultaneously non-judgmental, critical, and supportive of the government; (c) responses that supported the government; and (d) answers in which the respondent said they did not know about government pandemic responses. We present the result of this analysis in Table 6.

Study participants also provided open-ended responses to describe how they responded to the pandemic. Respondents typically described several changes they made on their farms or strategic shifts they made. We coded each unique pandemic response into a separate category. Table 9 contains a list of all the categories.

## Results

### Farm locations

This article analyzes 90 farms. Eighty (88.9%) are in the state’s Lower Peninsula; ten or 11.1% of the farms are in the Upper Peninsula. The farms tend to be in rural townships; they also hug the “fruit belt” along the Eastern shores of Lake Michigan.

### Demographic characteristics of farm owners and operators

We collected information on primary and secondary farm owners and operators. Hence, we collected data on 144 farm owners/operators. The majority, 88.2% (127), of the owners/operators were White, and 11.8% or 17 of the owners/operators were People of Color (Table 1). Eighty of the 127 (63%) of the White farm studied are primary owners/operators, while 37% (47) are secondary owners/operators. On the other hand, Farmers of Color are more likely to be secondary than primary farm owners/operators. Ten of the 17 (58.8%) Farmers of Color are primary owners/operators, and seven (41.2%) are secondary owners/operators.

**Table 1:** Demographic characteristics of farm owners/operators

Characteristics	Total owners/operators		Primary owners/operators		Secondary owners/operators	
	Number (*n* = 144)	Percent	Number (*n* = 90)	Percent	Number (n = 54)	Percent
*Race or ethnicity*						
White	127	88.2	80	88.9	47	87.0
*People of Color*	17	11.8	10	11.1	7	13.0
Sex						
Male	78	54.2	55	61.1	23	42.6
Female	66	45.8	35	38.9	31	57.4
*Work on the farm*						
Works full time	85	59.0	63	70.0	22	40.7
Works part time	55	38.2	27	30.0	28	51.9
Does not work on the farm at all	4	2.8			4	7.4

	Number	Mean age	Number	Mean age	Number	Mean age
*Mean age (in years)*	144	53.9	90	53.4	54	54.7
White	127	54.7	80	54.7	47	54.5
*Person of Color*	17	48.0	10	42.7	7	55.6
Male	78	54.7	55	55.3	23	53.2
Female	66	52.9	35	50.4	31	54.7

Males comprise 54.2% (78) of all the owners/operators. However, they are more likely to be primary owners/operators than secondary ones. Hence, 61.1% (55) of the primary owners/operators are male. In contrast, females account for 57.4% (31) of the secondary farm owners/operators.

Most farmers work on the farm full time; 59% (85) do. Moreover, 70% (63) of the primary owners/operators are full-time employees of their farm. Only 40.7% (22) of the secondary owners/operators work on their farms full time. The data show that 30% (27) of the primary owners/operators and 51.9% (28) of the secondary owners/operators work on the farms part-time. Only four farm owners/operators do not work on the farm, and all are secondary owners/operators.

The mean age for the farm owners/operators is 53.9 years. The primary owners/operators are slightly younger than the secondary owners/operators. While the mean age for the primary owners/operators is 53.4 years, it is 54.7 years for the secondary owners/operators. White farm owners/operators tend to be older than People of Color farm owners/operators. The mean age for the White owners/operators is 54.7 years and 48 years for People of Color owners/operators. The biggest age gap is apparent between White primary owners/operators and People of Color owners/operators. On average, White primary farm owners/operators are about 12 years older than the primary People of Color farm operators/owners. The People of Color who are primary farm owners/operators also tend to be younger than secondary owners/operators. While the mean age for People of Color primary farm owner/operator is 42.7 years, it is 55.6 years for secondary farm owners/operators.

Males who were primary farm owners/operators tended to be older than females who were primary owners/operators. The mean age for the male primary owner/operators was 55.3 years. The mean age of their female counterparts was 50.4 years. The opposite is true for secondary farm owners/operators. Female secondary owners/operators have a slightly higher mean age than males who are secondary owners/operators of farms. While males who are primary owners/operators are 2.1 years older than the males who are secondary owners/operators, the age difference amongst the female primary and secondary owners/operators is larger. The mean age of female primary farm owners/operators is 50.4 years. The females who are secondary farm owners/operators are, on average, 4.3 years older; their mean age is 54.7 years.

### Farm characteristics and the race/ethnicity of owners and operators

White farmers tend to operate larger farms than People of Color owners/operators. The most common farms in our study are less than 20 acres; 41.1% (37) of the farms are 1–19 acres in size (Table 2). Almost a third (29) of the farms are 20–99 acres in size, and 26.7% (24) of the farms studied are 100 acres or larger. Most People of Color owners have farms that are less than 20 acres in size; that is, 86.7% (13) of People of Color owners/operators oversee farms that are 1–19 acres. In comparison, 32% (24) of Whites operate similarly sized farms. That is, 51 (68%) of Whites operate farms that are 20 acres or more. While the mean farm size of White owners/operators was 62 acres, it was 12.1 acres for People of Color owners/operators.

**Table 2:** The relationship between farm characteristics and the race/ethnicity of owners/operators

Characteristics	Total sample		White		People of Color	
	Number	Percent	Number	Percent	Number	Percent
*Size of farm*						
1–19 Acres	37	41.1	24	32.0	13	86.7
20–99 Acres	29	32.2	28	37.3	1	6.7
100 Acres or More	24	26.7	23	30.7	1	6.7
*Number of owners/operators*						
One	36	40.0	28	37.3	8	53.3
Two	54	60.0	47	62.7	7	46.7
*Number of full or part-time workers*						
None, no answer	36	40.0	31	41.3	5	33.3
1–9	34	37.8	26	34.7	8	53.3
10 or more	20	22.2	18	24.0	2	13.3
*Ownership of the farm*						
Operator owns all of the farm	54	60.0	44	58.7	10	66.7
Operator rents, leases, or other	36	40.0	31	41.3	5	33.3
Arrangements for some/all of the farm						
*Organic or conventional*						
Certified organic	13	14.4	12	16.0	1	6.7
Uses organic practices but not certified organic	48	53.3	35	46.7	13	86.7
Some certified organic and some not certified organic	4	4.4	3	4.0	1	6.7
Conventional; not organic	25	27.8	25	33.3	0	0.0
*Length of time operating the farm*						
1–9 years	23	27.4	16	22.9	7	50.0
10–19 years	31	36.9	26	27.1	5	35.7
20 or more years	30	35.7	28	40.0	2	14.3

Typically, two owners/operators oversee the farm—60% (54) of the farms have a primary and a secondary owner/operator. However, there are striking differences between White-owned/operated farms and those owned/operated by People of Color. Two farmers (primary and secondary) oversee almost 63% (54) of the White-owned/operated farms. In contrast, only 46.7% (seven) of the People of Color farms have two farmers overseeing the operations.

The farms usually have a small workforce. Forty percent (36) of the farms have no workers or did not answer the question, while 34 (37.8%) have between one and nine workers. Twenty farms (22.2%) had ten or more employees. White-owned/operated farms tend to have a larger workforce than those operated by People of Color. While 24% (18) of White-run farms have ten or more workers, only two (13.3%) People of Color owned/operated farms have as many workers.

There is a high rate of ownership of the farms studied. The current operators owned 60% of the farms. Only 40% (36) of the operators rented, leased, or had other financial arrangements with their farms. About 59% (44) of White farmers and two-thirds (ten) of People of Color farmers own the farms they operate.

Most of the farms studied used organic practices. Only 27.8% or 25 farms were wholly conventional. Most farms (53.3% or 48) used organic methods, but they did not have organic certification. That is, they did use organic techniques on the farms. However, 13 (14.4%) of the farms in our study were certified organic. Another 4.4% (four) had mixed organic practices; the farmers used organic methods on the whole farm, but only some of their products had an organic certification.

Most People of Color (86.7% or 13) operate farms that use organic practices, but they do not have organic certification. In comparison, 46.7% (35) of White farmers use organic farming practices, but they do not have organic certification. More specifically, 16% (12) of White farmers have a farm with organic certification, so do 6.7% (one) of Farmers of Color own or operate such farms. Though a third (25) of White farmers owned/operated conventional farms, no People of Color owned or operated conventional farms.

The farms were usually well established. While 23 (27.4%) of the owners operated their farms for less than a decade, 61 (72.6%) operated for ten or more years. Thirty of the owners (35.7%) worked their farms for 20 or more years. However, People of Color have much shorter tenure on their farms than White farmers. Seven (50%) of the People of Color farmers operated their farms for 1–9 years; 16 (22.9%) of the White farmers operated for less than ten years.

### Farm financing and racial/ethnic characteristics of owners/operators

Most farmers indicate they have not taken out any loans to finance their farms; 46 out of 73 (63%) of the farmers who answered this question have not taken out farm loans. However, 37% (27) of the farmers have taken out loans (Table 3). The data show that more farmers seek loans than succeed in taking out loans for their farms. While 27 (37%) farmers have taken out farm loans, 40 (69%) said they tried to obtain loans in the past decade. More specifically, 25 (43.1%) of the farmers tried to take out one to three loans, and another 15 (25.9%) tried to get between four and ten loans for their farms.

**Table 3:** Race/ethnicity of farm owners/operators and farm financing activities

Financing activities	Sample		Sought financing				Obtained financing				Rate of loan approval	
			White		People of Color		White		People of Color		White	People of Color
	Number	Percent	Number	Percent	Number	Percent	Number	Percent	Number	Percent	Percent	Percent
Have taken out loans to finance farm operations	27	37.0	23	37.1	4	36.4						
Have had difficulty obtaining loans to finance farm	24	40.0	19	37.2	5	55.6						
*Number of farm loans sought in the past decade*												
None	18	31.0	16	32.0	2	25.0						
1–3	25	43.1	22	44.0	3	37.5						
4–10	15	25.9	12	24.0	3	37.5						
*Where have farmers sought and obtained loans from in the past decade:*												
Farm Service Agency	21	31.3	18	31.0	3	33.3	15	30.6	0	0.0	83.3	0.0
Banks	18	26.5	16	26.7	2	25.0	11	22.0	2	25.0	68.8	100.0
Family members	15	22.1	13	22.0	2	22.2	13	26.5	2	22.2	100.0	100.0
Credit unions	12	18.5	6	10.5	6	75.0	3	6.5	4	50.0	50.0	66.7
Farm Credit Services of America	11	17.2	10	17.9	1	12.5	10	21.3	1	12.5	100.0	100.0
Private investors	10	14.7	8	13.6	2	22.2	8	16.7	2	22.2	100.0	100.0
Cooperatives	4	6.2	4	7.0	0	0.0	4	8.5	0	0.0	100.0	0.0
Friends and acquaintances	4	6.1	3	5.3	1	11.1	3	6.7	1	11.1	100.0	100.0

Twenty-three (37.1%) Whites four (36.4%) People of Color took out loans to finance their farms. However, five of the nine (55.6%) People of Color farmers answering this question reported difficulty obtaining loans. Nineteen of 51 (37.2%) White farm owners/operators who responded to this question also said they had difficulties obtaining loans for their farms.

Farmers are most likely to seek financing from the FSA, an arm of the USDA. Twenty-one (31.3%) farmers sought FSA loans, and 15 succeeded in getting them. All the farmers who got FSA loans were White. So, 15 of the 18 or 83.3% of White farmers who applied for FSA loans succeeded in getting the loans. In contrast, none of the three People of Color farmers who applied for FSA loans got them.

People of Color farmers were more successful in obtaining credit union loans; four of the six (66.7%) People of Color farmers who applied for credit union loans obtained them. The two Farmers of Color who applied for bank loans also got them. In comparison, three of the six (50%) White farmers who sought credit union loans got them, and 11 of the 16 (68.8%) who applied for bank loans succeeded in getting them.

While four White farmers obtained loans from cooperatives, no Farmer of Color sought loans from this type of institution. Farmers also turned to other sources for loans; they sought and got loans from family, friends, private investors, and the Farm Credit Services of America.

Farmers also reported specific challenges they faced while seeking financing. Sixteen farmers—13 White and three People of Color—said they did not borrow any money to finance their farms. Another 11 farmers reported not encountering any difficulties when seeking loans. However, most farmers who borrowed money said they faced challenges when seeking financing for their farms. Table 4 summarizes the themes that arose when farmers described their experiences during the financing process.

**Table 4:** Race/ethnicity of farm owners/operators and challenges obtaining loans

Factors identified	Total	White owners/operators	People of Color owners/operators
Do not borrow or try not to borrow	16	13	3
None, not applicable	11	9	2
Difficulty with the Farm Service Agency—not farmer friendly loan system	5	4	1
High debt to equity ratio a hindrance, not enough income to secure loan	4	3	1
Low credit score	3	2	1
Commercial loans with very unattractive terms are offered	2	2	
Charged interest rates that are too high	2	2	
Uncertainty of the future of farming	2	2	
Large amount of paperwork	2	2	
Do not own enough land, too small	2	2	
Options are limited	1	1	
Farms are not recognized by conventional lenders	1	1	
Difficult to get loans or qualify for loans	1	1	
Large amount of equipment or land asked for as collateral	1	1	
Farm improvements delayed because of difficulty getting loans	1	1	
Financial institutions are reluctant to lend operating capital	1	1	
Have to take out personal loans to finance business	1	1	
Making accurate predictions of costs for the business plan	1	1	
Don't know the system	1	1	
Grants are better than loans	1		1
Personal loans are better than bank loans	1		1
Tried to refinance mortgage but was unsuccessful	1		1
Have to have off-farm job to have enough income to qualify for loan	1		1
Profitability during COVID is difficult	1	1	
Assets are undervalued	1	1	
Not yet making a profit	1	1	
Organizations do not want to help	1		1
It takes too long to process loans	1	1	
Too new to obtain a loan	1	1	
The credit union does not lend for agricultural purposes	1	1	
Did not have collateral necessary to secure loans	1	1	
Take family loans instead	1	1	

For instance, four White farm owners/operators and one Person of Color operator said that the FSA was not a farmer-friendly loan system. Other farmers noted that the terms of the loans were unattractive and that the interest rates were too high for them to afford. Five farmers indicated that they paid rates as high as 10%. Three other farmers paid 7% interest, while five paid 6%. Twenty-three farmers had loans with interest rates between 2 and 5%.

### Availability of funding during the pandemic

Farmers identified where they obtained financial assistance during the pandemic. White farm owners/operators accessed a more comprehensive range of financial assistance programs than Farm Owners/Operators of Color. Hence, White farm owners/operators reported that they accessed 15 sources of financial assistance while People of Color farm owners/operators reported receiving financial assistance from only three sources (Table 5). Thus, none of the People of Color farm owners/operators obtained paycheck-protection program (PPP) loans. People of Color farm owners/operators did not receive any CFAP-1 loans either. However, one of the seven CFAP-2 loans went to a Person of Color farm owner/operator. Four Farmers of Color received stimulus checks and one received unemployment benefits. Neither the stimulus checks program nor unemployment benefits are explicitly targeted to farmers per se; they are assistance geared towards the general population.

**Table 5:** Race/ethnicity of farm owners/operators and financial assistance during the COVID-19 pandemic

Source of funding	Total	White owners/operators	People of Color owners/operators
None	28	22	6
Paycheck Protection Program (PPP) loans	11	11	
Coronavirus Food Assistance Program 1 (CFAP)	10	10	
Coronavirus Food Assistance Program 2 (CFAP)	7	6	1
Federal government stimulus check	7	3	4
Personal Protective Equipment (PPE) loans	4	4	
U.S. Department of Agriculture funding	2	2	
Economic Injury Disaster Loans (EIDL)	2	2	
Small Business Administration loan	2	2	
Unemployment benefits	2	1	1
Food Assistance Lunch Box Program	1	1	
Small grants	1	1	
Private foundation grant	1	1	
Grain subsidies	1	1	
Chamber of Commerce gift	1	1	
Michigan Economic Development Corporation (MEDC)	1	1	

#### Response to government assistance

Study participants indicated how they felt about the government’s efforts to assist farmers. A total of 61 farm owners/operators stated how they felt about the government’s response to the pandemic (Table 6). While 25 (41%) were critical of government responses, 18 (29.5%) of the farmers were supportive of what the government had done to respond to the pandemic, and 13 (21.3%) were ambivalent. Eighteen out of 50 (36%) White farm owners/operators were critical of the government responses, so were seven of eleven (63.6%) People of Color farm owners/operators responding to the question. Though none of the Farm Owners/Operators of Color supported the government responses, 18 (36%) White farm owners/operators expressed approval.

**Table 6:** Race/ethnicity of farm owners/operators and perceptions of government responses to the COVID-19 pandemic

Perceptions of government responses to the pandemic	Total (*n* = 61)		White owners/operators (*n* = 50)		People of Color owners/operators (*n* = 11)	
	Number	Percent	Number	Percent	Number	Percent
Critical of government responses	25	41.0	18	36.0	7	63.6
Ambivalent about government responses	13	21.3	11	22.0	2	18.2
Supportive of government responses	18	29.5	18	36.0	0	0.0
Don't know about government responses	5	8.2	3	6.0	2	18.2

#### Criticism of government responses

Fifty-six respondents wrote detailed comments about government responses to the pandemic. Twenty-five respondents wrote critical comments. The criticisms fell into two thematic areas: the belief that the government favored large farmers over small ones in assistance programs and the feeling that the response was slow and cumbersome. The following discussion uses quotes from farmers’ open-ended responses to illustrate each theme identified.

##### Favoring large farmers over small farmers

Favoritism was a point of contention for some study participants who criticized government responses during the pandemic. A White female farmer from Southwest Michigan said of the government, “Well, they help large farmers who export, but for small farmers like us, we don’t believe they’ve done anything.” The small farmers were troubled by what they saw as efforts made to help large producers while ignoring small producers.

A White farmer from the Upper Peninsula of Michigan echoed this theme in his comments. He noted that “CARES [Coronavirus Aid, Relief, and Economic Stability Act] funding did not help the small farmer [and] most of it went to larger farms.” Additionally, another said, “I think it’s far past due that the government actively supports small farms.” A farmer also said, “I think farmers, like many small businesses (especially restaurants), are being left behind.”

Farmers thought that the distribution of CFAP-1 was unfair. It is evident in statements like, “CFAP was a handout for larger farmers votes.” A Farmer of Color from Southeast Michigan reported that after small farmers saw how CFAP-1 was disbursed, they organized, expressed their concerns, and advocated for change. The farmer noted,

The first ag[riculture] COVID relief package [CFAP-1] didn't apply to us, which was extremely frustrating. They did fix most the issues that farmers like [me] brought up.

A White organic farmer from Southeast Michigan also criticized the emergency assistance to large farmers because he felt that CFAP ignored small farmers using sustainable practices. The farmer said,

They are creating an unfair advantage to those that are getting the funding and hurting other farmers who… don't qualify for the funding…The government subsidies and [is] financing unsustainable farming operations that would not be able to support themselves without it…Farms like mine who are producing organic and sustainable products have to compete for resources (farmland, equipment, etc.) with farms that are getting government funding, and it gives them an unfair advantage that they wouldn't have on a free market.

Some farmers see the lack of consideration of small farmers in the early round of aid assistance as a sign of disrespect. Reflecting on this, a White female farm owner/operator from Central Michigan said,

...Small farmers are not respect[ed] through the USDA or MDARD [the Michigan Department of Agriculture & Rural Development]. We are known as 'freaks' before the pandemic, so what do you think they think of us now.

Others pointed to the back-and-forth between the state and the federal governments as problematic. According to a White male owner/operator from the Southeast portion of the state, the "state of Michigan didn't do much for farmers, but it is not their job, the federal government did a very poor job." Farmers were also frustrated by the stalemate between the Republicans and Democrats. As one White female owner/operator from the Southwestern part of Michigan saw it,

…they did the first (CARES Act) but have been arguing between the parties (GOP [Republicans] and DEM [Democrats]) ever since while people remain unemployed, going through savings, etc. That makes it difficult for them [the customers] to make higher purchases at farmers markets etc.

##### Slow and inadequate government response

Study participants also mentioned the speed of the response and the clarity of the guidelines under which they and farmer's markets could operate. A White female farm owner/operator from Southwest Michigan said the response was "slow, very hard to navigate, focused on large commodity producers, uninformed and unfocused." A female Farm Owner/Operator of Color from South Central Michigan commented on the federal and state response. She felt the government's response was,

Very poor. Our local health dep[artmen]t and the state are doing the best they can, but the Federal response is lousy.

While one farmer said the response was "terrible for a[n] owner/operator of a small farm," another said,

I wish they would have been a lot quicker setting out specific guidelines for how farmer's markets should operate when the pandemic first started. There was a lot of confusion and inconsistency across markets in practices; some stayed open, some closed, and there was no cohesive strategy for making a market safe. This affected us [because] it is our main place of sales.

A White female respondent from Central Michigan thought that the government was unprepared to address the crisis. She notes,

I think the response, in general, was woefully inadequate, but especially for small farmers. The CFAP-2 assistance made it easier for small farmers to get some relief. Overall, though, it was clear that the government had not fully prepared for how to address such a crisis.

Another White female study participant from Central Michigan who said the government's response was "bad" explained that,

The government's response has been a through-line of disappointment and death…we needed leadership. Farmers are resilient people who didn't expect or receive special assistance.

#### Ambivalence

Thirteen farmers were non-judgmental about or did not react to the government’s financial assistance to farmers. Others saw both negatives and positives in government responses. Statements like this, which came from a White female farm owner/operator in the Upper Peninsula, exemplify the ambivalence.

The government did what it could, but dairy is a specialty product. We cannot sell it anywhere but thru the coop to a Grade A processor, so our hands are tied on price and markets.

The following statement by a White Upper Peninsula farm owner/operator is also ambivalent. He thought the government’s assistance was,

Probably overly generous. Glad to see that [the government] spread the help around to more than just the corn, soybean, and cotton farmers.

Another farm owner/operator—a White female from Central Michigan—questioned the necessity of government assistance and who received aid in this statement,

It [the assistance] was great but, likely unnecessary. BIPOC [Black, Indigenous, and People of Color] and LGBTQ [lesbian, gay, bisexual, transgender, and queer] farmers deserve more support but, the broad strokes of the loan program were probably not necessary. If those with the most opportunity and generational wealth cannot survive through this and adapt, I do not feel sorry for them, nor should they have been given grants.

#### Praise for the government’s handling of assistance to farmers

Eighteen farmers were supportive of the government’s pandemic responses. They used phrases like “great” and “excellent” to praise the government. A White male farmer from Southwest Michigan said, “The COVID grant to our farm made a substantial difference.” A White female farm owner/operator from the Northwest portion of the Lower Peninsula said, “It has been good. The programs we applied for had simple applications and quick responses.” Others reported that the assistance was “very helpful” and “better than expected. We are thankful for anything we received.” A White female Southeast Michigan farm owner/operator also said,

We are very much proponents of the CFAP payments to farmers as well as funding for food distribution to food banks.

Another White female study participant from the Upper Peninsula reiterated this point by saying that “…allowing farmers to participate in food banks and other forms of food assistance was a great idea.” A White male respondent from Southwest Michigan who thought that government officials “were fairly responsive” also said that such officials “tried to make the process fairly easy. I appreciate[d] the help.” One farmer—a White male from the Northwest part of the Lower Peninsula—concluded by saying, “So far so good, it [the government assistance] has kept us in business.”

### Impacts of the pandemic on farming operations

#### Farming and sales

The pandemic impacted the farmers studied in many ways in 2020. Seventy-one farmers answered the question about the start of the growing season. Forty-nine (69%) of them said they started farming at the same time as usual in 2020. However, 16 (22.5%) indicate that they had to delay their farming activities because of COVID-19. Hence, 11 of 60 (18.3%) White farmers and 5 of 11 (45.5%) People of Color farmers started their farming activities later than usual. Six or 8.5% of the farmers started farming activities earlier than usual. Though our study inquired only about the impacts of COVID-19 on the start of farming activities for the year, other factors such as unseasonably cold weather, flooding, lack of workers, and financial troubles can also cause delays.

As Table 7 shows, 79.3% (46) of White farm owners/operators and all the People of Color farm owners/operators said they farmed the same number of acres as they did the previous year. However, 19 farm owners/operators (15 White and four People of Color) said they had fewer farmers’ markets to sell their products. In other words, 28.8% of White farm owners/operators and 44.4% of Farmers of Color found this to be the case.

**Table 7:** Race/ethnicity of the farm owners/operators and the impacts of the pandemic on farm operations in 2020

Impacts	Total sample				White farm owners/operators				People of Color farm owners/operators			
	Number	Decreased	Remained the same	Increased	Number	Decreased	Remained the Same	Increased	Number	Decreased	Remained the Same	Increased
The number of acreage farmed	68	7.4	82.4	10.3	58	8.6	79.3	12.1	10	0.0	100.0	0.0
The number of customers buying our produce	66	25.8	19.7	54.5	57	24.6	17.5	57.9	9	33.3	33.3	33.3
The amount of revenue we generate from the farm	65	32.3	32.3	35.4	56	30.4	33.9	35.7	9	44.4	22.2	33.3
The number of workers we have	62	33.9	50.0	16.1	53	30.2	54.7	15.1	9	55.6	22.2	22.2
The number of volunteers we have	62	37.1	53.2	9.7	51	31.4	58.8	9.8	11	63.6	27.3	9.1
The number of open farmers market that we could sell produce at	61	31.1	65.6	3.3	52	28.8	69.2	1.9	9	44.4	44.4	11.1
The amount of funding we have to conduct farming activities	61	24.6	47.5	27.9	52	25.0	46.2	28.8	9	22.2	55.6	22.2
The number of people seeking food assistance from the farm	55	10.9	69.1	20.0	46	10.9	71.7	17.4	9	11.1	55.6	33.3
The amount of produce we supply restaurants with	54	46.3	50.0	3.7	45	48.9	48.9	2.2	9	33.3	55.6	11.1
The number of other institutions we can sell our products at	54	31.5	63.0	5.6	47	36.2	59.6	4.3	7	0.0	85.7	14.3
Our community supported agriculture operations	48	18.8	50.0	31.3	40	17.5	47.5	35.0	8	25.0	62.5	12.5

The urban or rural location of the farmers market might influence if or when it opened during the pandemic. Sixteen (17.8%) of the 90 farms studied were in urban areas, and seven (9.3%) of the 75 White farmers operated farms in urban areas. In comparison, 9 (60.0%) of the 15 People of Color farmers operated farms in cities. Most of the urban farms included in this study were in the southern part of the state. The pandemic ravaged Southeastern Michigan first, then slowly spread northwards and westwards during the late summer. Therefore, it is likely that the pandemic could have caused farmer’s markets in Southern Michigan to close.

When restaurants and other institutions closed, it also reduced the number of venues the farmers studied could sell their products. A higher percentage of White farm owners/operators reported not selling to restaurants and other institutions than People of Color farm owners/operators. Twenty-two (48.9%) White farm owners/operators said they sold to fewer restaurants because of the pandemic. In comparison, three (33.3%) People of Color farm owners/operators reported that their sales to restaurants declined. White farmers were also more likely than Farmers of Color to report that the number of other institutions they sold products to decreased; 17 (36.2%) of White farmers and none of the Farmers of Color said this was the case.

In 2020, most White farm operators hired roughly the same number of workers they had in 2019 and mobilized roughly the same volunteers. However, this was not the same for Farmers of Color. Twenty-nine (54.7%) of 53 White farm owners/operators hired roughly the same number of workers as the previous year. However, only two (22.2%) of nine Farmers of Color hired the same number of workers. Five (55.6%) of the Farmers of Color said they hired fewer workers than the previous year. Farmers of Color also reported that the number of volunteers who helped on their farms shrunk in 2020; seven of eleven, or 63.6%, said this was the case.

Farmers in our sample reported an increase in some of their farming, marketing, and sales activities. For instance, 16 (35%) White farmers and one (12.5%) Farmers of Color increased their community-supported agriculture (CSA) activities. Farmers also reported that they had more customers in 2020 than in 2019. White farmers were more likely to report increased customers than Farmers of Color. Hence, 33 (57.9%) White farmers and three (33.3%) Farmers of Color said more customers purchased items from them in 2020. Overall, 35.4% (23) of the farmers studied reported that the revenues generated from the farm increased in 2020. Farmers also saw an increase in the number of people seeking food assistance from their farms; this was particularly true at farms operated by People of Color. Thus, 17.4% (8) of White owners/operators and a third (3) of the People of Color owners/operators reported increased requests for food assistance.

#### The fate of unsold products

Food insecurity increased dramatically because of the pandemic, and farmers are critical actors to meet the demand for emergency food (Benton 2020; Dickinson 2020; Emmad and Peña 2020). Consequently, we asked farmers to say what they did with produce and other perishable items that they did not sell. Farmers frequently gave away unsold products to their friends, family, or neighbors; 82.6% (57) did this. All eleven Farm Owners/Operators of Color and 46 (79.3%) White farm owners/operators gave food to family, friends, or neighbors (Table 8).

**Table 8:** Race/ethnicity of owners/operators and what happens to unsold farm products

Fate of unsold farm products	Total sample		White		People of Color	
	Number	Percent	Number	Percent	Number	Percent
Give them away to friends, family, or neighbors	57	82.6	46	79.3	11	100.0
Compost them	45	65.2	35	60.3	10	90.9
Feed them to farm animals	42	59.2	36	60.0	6	54.5
Sell them at reduced prices	37	52.9	31	52.5	6	54.5
Give them away to anyone who asks	37	52.9	27	45.8	10	90.9
Preserve them	36	51.4	31	52.5	5	45.5
Invite people to glean it	34	48.6	25	42.4	9	81.8
Plough it back into the fields for fertilizer	33	47.1	26	44.1	7	63.6
Donate them to food pantries	32	47.1	27	47.4	5	45.5
Donate them to soup kitchens	20	29.0	15	25.9	5	45.5
Donate them to food aggregators	20	28.6	17	28.8	3	27.3
Throw them away, dispose of them	20	28.6	18	30.5	2	18.2
Donate them to shelters	15	22.4	12	21.4	3	27.3
Donate them to churches/religious institutions	15	21.7	11	19.0	4	36.4
Donate them to schools	12	17.4	9	15.5	3	27.3

Twenty-seven (45.8%) of 59 White farm owners/operators said they gave away unsold food or perishable products from the farm to anyone who asks. Ten (90.9%) of eleven Farmers of Color did the same. Similarly, for gleaning, nine (81.8%) of the Farmers of Color said they invited people to glean their farms. In comparison, 25 (42.4%) White farm owners/operators invited people to glean on their farms.

Thirty-seven (52.9%) of the farmers said they reduced the price of products to sell them. Some of the study participants preserved unsold food while others threw it away. While 36 (51.4%) of the respondents said they preserved unsold food, 20 (28.6%) reported throwing such food away. Thirty-two farmers (47.1%) donated unsold products to food pantries. A smaller number, 20 (28.6%), donated unsold products to soup kitchens and food aggregators.

#### Other responses to the pandemic

During the pandemic, farm owners/operators made a variety of changes in the way they operated their farms or conducted their business. Table 9 contains the most common themes that emerged in the farmers' responses to the pandemic. The most frequently reported response to COVID-19 was to institute social distancing practices on their farms, at their farm stores and stands, or when they sold products at farmer's markets. Ten farmers reported they instituted social distancing practices, while another seven required customers to wear masks in their stores or on their farms. Five farmers focused on enhancing sanitation at their farms, while four installed handwashing and sanitizing stations. Farmers also said they communicated clearly with customers about safe practices on their farms, and they installed plastic shields to separate customers from cashiers and other employees.

**Table 9:** Race/ethnicity of farm owners/operators and responses to the COVID-19 pandemic

Responses	Total	White owners/operators	People of Color owners/operators	Responses	Total	White owners/operators	People of Color owners/operators
Practice social distancing; post social distancing instructions	10	8	2	Hired more employees to handle increased sales	1	1	
Added online storefront—as summer and year-round operation	8	7	1	Reconfigured customer traffic flow at the farm	1	1	
Customers and staff have to wear masks in on-farm store, at the farm	7	6	1	No touching of products	1	1	
Nothing new—business as usual	6	5	1	Decrease food offerings from the kitchen	1	1	
Increased focus on sanitation at the farm	5	3	2	No hay rides or pony rides	1	1	
Stop or reduce selling at farmer's markets	4	4		Still keeping the focus on direct selling	1	1	
Make home deliveries of orders—contactless	4	4		Donating more products from the farm	1	1	
Install hand-washing and sanitizing station for customers	4	3	1	Limit the number of customers on the farm	1	1	
Trying to adjust to loss of customers	4	4		Opened a self-served farm stand at the farm	1		1
Trying to keep up with and adjust to the changing rules	4	4		Increased operating capital	1	1	
Increase our community supported agriculture (CSA) activities	3	3		Reduced debt	1	1	
Pick up orders at local farmer's markets—contactless	3	3		Curtail educational activities	1	1	
Pick up orders at the farm—contactless	3	2	1	Stop farm stays	1	1	
It is harder to process meat; taking the animals to more processors	3	2	1	Shift to growing microgreens	1		1
Make reservation meat processors well in advance; few are open	3	2	1	Purchase equipment to start growing indoors earlier	1		1
Increased the size of the on-farm, u-pick market	3	2	1	Streamlining water system	1		1
Identifying ways to sell livestock; difficulty selling farm animals	3	3		Identifying new customer base	1	1	
Increased sales	3	3		Shift to outdoor activities like apple picking	1	1	
Stressed, going crazy, barely coping	2	2		Organize staff training	1	1	
Increased purchase of livestock, increased the sale of meat	2	2		Make COVID testing available to workers	1	1	
Plastic barriers between customers and cashiers, employees	2	2		Seeds and plants were limited as more people grew gardens	1	1	
Communicate about safe practices at the farm	2	1	1	Trying to find canning supplies – there was a shortage	1	1	
Lowering prices, having sales	2	2		Shared seeds with neighbors	1	1	
Hold a volunteer day	2	1	1	Froze extra products	1	1	
Trying to find farm workers	2	2		Plant more	1	1	
Used distributor to sell products	2	2		Build greenhouse	1		1
Customers pick out and purchase bags of pre-packaged products	1	1		Update and upkeep the farm	1		1
Institute new practices	1	1		Did not hire any staff	1	1	
Re-organizing u-pick operations	1	1		Cut back on the amount planted	1	1	
Making concerted attempts to get chickens, farm animals to grow/process	1	1		Sold nursery plants to home gardeners	1	1	
Reducing costs by cutting down on the number of milking animals	1	1		Closed the farm	1	1	
Searching for and finding new opportunities	1	1		Canceled farm tours and school outings	1	1	
Forgoing needed things	1	1		Stop selling meat	1		1
Trying to make new business start-up (the farm) work	1	1		Reduced number of hours everyone worked	1		1
Increase in the number of customers	1	1					

Eight farmers (seven White and one Person of Color) moved some of their business online. Consequently, they expanded their sales by selling during the summer and year-round. Farmers also devised alternative forms of contactless deliveries to customers. Hence, four farmers made home deliveries, three allowed customers to pick up purchases at local farmer’s markets, and another three allowed customers to pick up purchases at the farms.

Farmers identified several challenges with obtaining chickens and other livestock they wanted to grow. Some said they had difficulty finding places to slaughter and process animals after the meat-processing facilities they were accustomed to using shuttered temporarily.

Farmers also said they had difficulty finding enough seeds or plants to purchase. It was the case because many more people started home gardens and grew their fruits and vegetables. In response, one farmer began selling nursery plants to home gardeners. Home gardeners also canned and preserved more than usual in 2020. It meant that farmers could not find enough cans and other materials needed to can, bottle, and preserve their products. Unable to secure enough cans, one farmer froze excess products in the hopes of using them in the future.

Farmers were nimble and continually shifted their strategies to take advantage of new market opportunities. One Farmer of Color reported that their farm installed a greenhouse and shifted to growing microgreens to meet the increased demand for the product and extend their growing and sales season.

Farmers also reorganized the u-pick activities on their farms. One created a self-serve farm stand, while others prepared pre-packaged bags of products that customers could select for purchase. Farmers also instituted a no-touch (the products) policy. One farmer reported stopping selling meat from their farm while another closed their farm. Some shifted to outdoor fruit-picking activities. Hence, some shifted to apple-picking or similar types of fruit picking that could utilize social distancing. Farmers also canceled educational and social activities, hayrides and pony rides, school tours, and farm stays.

## Discussion

This article is one of the first to examine how COVID-19 impacted farmers and farming operations. It is also unique in that it examines how farm owners/operators’ racial/ethnic characteristics influence farm finances, operations, and the impacts of the pandemic and its responses. The paper highlights crucial information about farmers that merit further scholarly inquiry.

Before COVID-19, the USDA found that farm sales declined (USDA 2019a, b, c, d, e). About a third of the study participants said that their revenues in 2020 were less than in 2019. Our data suggest that farm revenues might have declined further for some of the farmers in our study. Declining revenues is concerning as the farms owned/operated by People of Color might be vulnerable because they tend to be smaller and with precarious financing. We found that four of nine People of Color farm owners/operators reported a decline in revenues. Our findings are consistent with other studies, which find that Farmers of Color own/operate smaller farms than White farmers (Carlisle et al. 2019; Horst and Marion 2019; Taylor 2018).

The study also found that Farmers of Color had difficulty securing loans to enhance their farm operations and accessed a smaller pool of federal loan assistance programs during the pandemic than White farm owners/operators. Past studies found that USDA and the FSA have systematically discriminated against Black and other Farmers of Color for decades (Taylor 2018; Daniel 2013; Minkoff-Zern and Sloan 2017; Carlisle et al. 2019). Scholars also report that Black farmers in Michigan have difficulty obtaining loans from these sources (Tyler et al. 2014). None of the Farmers of Color in our study obtained an FSA loan.

Another concern arose as we found that government pandemic financial relief seemed to have bypassed some farm owners/operators in our study. While White farm owners/operators reported obtaining CFAP-1, CFAP-2, and other types of government grants and loans during the pandemic, only one Person of Color owner/operator reported receiving CFAP. Except for the stimulus checks and unemployment benefits that went to the general population, Farmers of Color were unlikely to report receiving the other kinds of financial support that White farmers received.

Several scholars have identified farm financial insecurity as a critical area that should concern us. Our findings lend credence to the claims of researchers who argue that more than half of all farmers are at risk of financial insecurity (Key et al. 2019; Hoppe 2017; Carlisle et al. 2019). Some farmers take off-farm jobs to make ends meet and stave off financial insecurity (USDA 2019d; Carlisle et al. 2019; Montri et al. 2021). We found evidence of this in our study. People of Color farmers are at increased risk of financial insecurity because their farms are small, have been operating for a short time, have declining sales, have young and inexperienced owners/operators, and have difficulty obtaining credit. Our findings suggest that we should pay more attention to Farm Owners/Operators of Color.

Farms owned/operated by People of Color might also be financially vulnerable for additional reasons. People of Color farms tend to have one owner/operator; more than half of their farms have one operator. It is in stark contrast to White-owned/operated farms—most of these have two owners/operators. The Farmers of Color in our study also own/operate farms that have fewer employees and volunteers than White-owned/operated farms. The size of the workforce has implications for expansion, adjusting to market changes like those brought on by COVID-19, and long-term viability. COVID-19 required farmers to assign more workers to make deliveries, shop for and pack customers’ orders, monitor customers to see that they follow social distancing rules, and process online orders.

Blay-Palmar et al. (2020) predict that farmers will start or expand their online sales in response to COVID-19, and our findings support this claim. However, the online market might be bypassing Farmers of Color; only one Farmer of Color reported selling their farm’s products online. Farmers who did not have the workforce to execute online-only or hybrid sales (online and in-person) were disadvantaged.

The FFFBP (Agricultural Marketing Service 2021; Galloway 2020; Sielski 2020) was popular with our study participants. The program provided a mechanism for farmers, who decided how much to grow before restaurants and other institutions they usually sold products to closed, to get their products to consumers. Through the program, farmers supplied soup kitchens, food banks, religious institutions, and other emergency food assistance nonprofits with produce to distribute to those seeking emergency food.

The FFFBP also arose because of rising food insecurity nationwide. The cost of food soared, and some foods were in short supply during the pandemic. It made it difficult for many people who lost their incomes to purchase food. Consequently, emergency food assistance programs were deluged with additional requests for food. Some food insecure individuals also sought food assistance directly from farmers. Fifty-five farm owners/operators said people sought food from them.

Some Farmers of Color may get requests for food because more of their farms are close to cities. In addition, the pandemic has affected urban Communities of Color disproportionately. There was evidence of food insecurity in such communities before the pandemic (Taylor and Ard 2015; Burdine and Taylor 2018). The emergency food requests may also be related to the expressed social mission of farms. Montri et al. (2021) found that some Michigan farmers say they enter farming to fulfill social missions. Such social missions include producing locally grown food, increasing food sovereignty in Communities of Color, or increasing access to healthy and affordable food. Some Farm Owners/Operators of Color publicly express social missions to reduce food insecurity in low-income and Communities of Color. Such messaging could result in increased requests for food during times of hardship.

Our findings suggest that food-seeking from farmers deserves further research attention. Farmers said they gave unsold produce and perishables to their friends, families, and neighbors. All the Farm Owners/Operators of Color reported that they did this. Farm owners/operators also gave away unsold produce and perishables to anyone who asked for it; they also allowed people to come to their farms and glean them. These findings suggest that farms may have social missions that are understudied.

## Conclusion

This paper suggests that we should pay more attention to small family farmers to find out how disruptions to the food system, like a pandemic, strain farm operations and provide opportunities to innovate. Though the closure of restaurants posed significant challenges to farmers, study participants showed nimbleness and resilience by instituting social distancing procedures at their place of business, offering new delivery options, and expanding their operations. Overall, some farm owners/operators were able to enhance the financial stability of their farms.

Farmers embraced the opportunity to increase their service to emergency food assistance programs. The pandemic provided an opportunity to connect farmers to customers through new programs such as the FFFBP. Farmers should nurture these relationships after the pandemic ends.

The study identifies crucial differences between farms operated by White owners and those operated by People of Color. The findings suggest that Farmers of Color are under extra stress from demands to give away food while they have difficulty financing their farms. Moreover, Farmers of Color do not report the same access to government pandemic assistance as their White counterparts. Though Farmers of Color have risen to the occasion and provided food assistance that they are not compensated for (like gleanings and food gifting), these aspects of their social mission could be putting them at greater risk for financial insolvency. The paper suggests that researchers and policymakers pay greater attention to the inequities to ensure that Michigan has a vibrant and diverse family farming sector.

## Abbreviations

BIPOC: Black, Indigenous, and People of Color
CARES: Coronavirus Aid, Relief, and Economic Stability
CFAP: Coronavirus Food Assistance Program
COVID: Coronavirus Disease
CSA: Community-supported agriculture
EIDL: Economic Injury Disaster Loans
FFFBP: Farmers to Families Food Box Program
FSA: Farm Service Agency
PPE: Personal Protective Equipment
PPP: Paycheck-protection program
LGBTQ: Lesbian, gay, bisexual, transgender, and queer
MDARD: Michigan Department of Agriculture & Rural Development
MEDC: Michigan Economic Development Corporation
USDA: United States Department of Agriculture

## Footnotes

1 Only 1.2% of U.S. farms are nonfamily farms. It means that the principal operator and those related to the primary operator do not own a majority share of the business (Hoppe 2017).

2 Midsize farms have gross cash farm incomes between $350,000 and $999,999; they comprise 6% of all farms. There are two categories of large-scale farms (constituting 2.9% of all farms): large farms have gross cash incomes of between $1,000,000 and $4,999,999, and very large farms have gross cash farm incomes of more than $5,000,000 or more (Hoppe 2017).
